# Vascular Adhesion Protein-1 (VAP-1)/Semicarbazide-Sensitive Amine Oxidase (SSAO): A Potential Therapeutic Target for Atherosclerotic Cardiovascular Diseases

**DOI:** 10.3389/fphar.2021.679707

**Published:** 2021-07-08

**Authors:** Hui Li, Shiyu Du, Panpan Niu, Xiaosong Gu, Jun Wang, Ying Zhao

**Affiliations:** ^1^ Department of Cardiology, The Second Affiliated Hospital of Soochow University, Suzhou, China; ^2^ Department of Pathophysiology, School of Biology and Basic Medical Sciences, Soochow University, Suzhou, China; ^3^ Institutes of Biology and Medical Sciences, Soochow University, Suzhou, China

**Keywords:** VAP-1, inflammation, atherosclerosis, myocardial infarction, stroke

## Abstract

Vascular adhesion protein-1 (VAP-1) is a semicarbazide-sensitive amine oxidase (SSAO), whose enzymatic activity regulates the adhesion/exudation of leukocytes in/from blood vessels. Due to its abundant expressions in vascular systems and prominent roles in inflammations, increasing attentions have been paid to the roles of VAP-1/SSAO in atherosclerosis, a chronic vascular inflammation that eventually drives clinical cardiovascular events. Clinical studies have demonstrated a potential value of soluble VAP-1 (sVAP-1) for the diagnosis and prognosis of cardiovascular diseases. Recent findings revealed that VAP-1 is expressed in atherosclerotic plaques and treatment with VAP-1 inhibitors alleviates the progression of atherosclerosis. This review will focus on the roles of VAP-1/SSAO in the progression of atherosclerotic lesions and therapeutic potentials of VAP-1 inhibitors for cardiovascular diseases.

## Introduction

Cardiovascular diseases (CVD) are the leading cause of death worldwide. Most CVD patients die of ischemic heart disease and ischemic stroke (Zhang et al., 2020). The pathological basis of these diseases is atherosclerosis, and the subsequent induction of vascular stenosis or thrombosis leads to clinical acute cardiovascular events. Ongoing pathological studies indicate that atherosclerosis is a chronic inflammatory reaction of the vascular wall in response to dyslipidemia and endothelial distress involving the infiltration of leukocytes and activations of resident vascular cells (Frostegård, 2013). The recruitment of immune cells into atherosclerotic lesions requires the expression of adhesion molecules on endothelial cells. Vascular adhesion protein 1 (VAP-1), encoded by the *AOC3* (amine oxidase copper-containing 3) gene, is an endothelial adhesion molecule with amine oxidase activity (Valente et al., 2008). The enzymatic activity of VAP-1 is highly sensitive to inhibition by semicarbazide (SCZ), and VAP-1 thus belongs to a family of semicarbazide-sensitive amine oxidase (SSAO). More and more evidence shows that VAP-1/SSAO is implicated in vascular diseases, probably due to the induction of leukocyte trafficking and vascular damage (Boomsma et al., 2005). This article reviews the involvement of VAP-1/SSAO in atherosclerotic diseases, including stroke and coronary artery disease (CAD), and discusses its role in atherosclerosis as well as the therapeutic potential of VAP-1 inhibitors for CVD.

## VAP-1: An Inducible Adhesion Molecule in Inflammation

VAP-1/SSAO is expressed in the vascular system, especially on the surface of endothelial cells, and regulates the adhesion and migration of circulating immune cells (Salmi and Jalkanen, 2019). A variety of *in-vitro* binding experiments have shown that bindings of lymphocytes, monocytes, and granulocytes to high endothelial venules (HEVs) and/or blood vessels in different tissues are partially dependent on the expression levels of VAP-1 (Salmi et al., 1997a; Salmi et al., 1997b; Salmi et al., 1998; Jaakkola et al., 2000). Real-time imaging shows that VAP-1 mediates the slow-rolling, firm adhesion and migration of leukocytes in blood vessels of lymphoid tissues and inflammatory sites (Stolen et al., 2005). Despite lowly expressed in a subset of venules of normal nonlymphatic tissues like skin, liver, and hearts, VAP-1 is abundant in HEVs of peripheral lymph nodes where the sialic acid residues of VAP-1 interact with counter-receptors on lymphocytes and thereby mediate their homing (Salmi and Jalkanen, 1996; Smith et al., 1998). During inflammation, endothelial VAP-1 is upregulated and its enzymatic activity is indispensable for leukocyte extravasation through endothelium (Salmi and Jalkanen, 2005). Accordingly, mice with *Aoc3* deficiency or mutant *Aoc3* lacking functional SSAO activity have defects in leukocyte migrations (Noonan et al., 2013). Moreover, inactivation of VAP-1/SSAO by specific inhibitors or neutralizing antibodies significantly reduces a variety of acute and chronic inflammatory diseases in experimental animals (Salmi and Jalkanen, 2019). The enzymatic actions of VAP-1 on leukocyte trafficking appear to be mediated by the production of hydrogen peroxide (Salmi and Jalkanen, 2019). Notably, apart from impaired leukocyte infiltration into inflammatory sites, decreased homing of immune cells to lymphatic organs due to VAP-1 deficiency/inactivation may also attenuate inflammations by inhibiting their activation and proliferation (Koskinen et al., 2007).

Although highly expressed, VAP-1 in both smooth muscle cells (SMCs) and adipocytes does not mediate lymphocyte binding (Jaakkola et al., 1999; Boomsma et al., 2005; Bour et al., 2009). In SMCs, VAP-1 is enriched in caveolae of the plasma membrane, but its physiological functions remain unknown (Salmi and Jalkanen, 2019). In contrast, the expression of VAP-1 is upregulated in adipocytes during differentiation or after incubation with the inflammatory cytokine TNF-α (Abella et al., 2004). In adipocytes, VAP-1 colocalizes with glucose transporter GLUT4 in an endosomal compartment, and its substrates stimulate the recruitment of GLUT4 to the plasma membrane, thereby promoting the uptake of glucose (Enrique-Tarancón et al., 1998; Yu et al., 2004). These insulin-like effects (i.e., increased glucose uptake and lipogenesis) of SSAO substrates on adipocytes require hydrogen peroxide generated in VAP-1’s enzymatic reactions (Zorzano et al., 2003). The potentials of adipose VAP-1 in the management of blood glucose/lipids and obesity thus have received special attentions. Interestingly, deletion of VAP-1 or expression of mutated VAP-1 lacking SSAO activity favors fat deposits in mice on regular chow diet without affecting food intake and glucose handling (Jargaud et al., 2020).

## Soluble VAP-1: A Potential Biomarker of CVD

Soluble VAP-1 (sVAP-1) in circulation is produced by proteolytic cleavage of its membrane-bound form (Abella et al., 2004; Stolen et al., 2004b), and plasma levels of sVAP-1 vary in different species (Boomsma et al., 2003). In humans, plasma sVAP-1 concentrations are nicely correlated to SSAO activities in both healthy controls and patients with various diseases (Boomsma et al., 2003; Aalto et al., 2012). Data obtained in mice specifically expressing human VAP-1 (hVAP-1) in different cells revealed that plasma sVAP-1 is mainly derived from endothelial cells under both physiological and inflammatory conditions, with some contributions of adipocytes and SMCs (Göktürk et al., 2003; Stolen et al., 2004b). However, whether the same holds true in humans remains to be resolved. Physiologically, organs like livers, kidneys, and legs are unlikely to be the major source of plasma sVAP-1 in humans, as concentrations of sVAP-1 in arteries and veins of these organs are comparable (Boomsma et al., 2003). We speculate that a substantial amount of sVAP-1 could be produced by HEVs of lymphatic organs, where matrix metalloproteinases (MMPs) required for transendothelial migration of lymphocytes may cut off the membrane-bound VAP-1. In chronic inflammatory liver diseases, higher levels of sVAP-1 in hepatic veins vs. portal veins indicate that inflammation may trigger the production of sVAP-1 by local endothelial and parenchymal cells in inflammatory organs (Kurkijärvi et al., 2000). Conversely, surgical correction of coronary atherosclerotic lesions leads to the lowering of elevated plasma SSAO activities (Boomsma et al., 2003).

Elevated circulating sVAP-1 levels are evident in patients with all kinds of CVD including CAD, arterial stiffness, aortic stenosis, hypertension with echocardiographic alterations, chronic/congestive heart failure, and ischemic/hemorrhagic stroke (Table 1) (Chen et al., 2015). Consistent elevation of plasma sVAP-1 also reflects the severity of congestive/chronic heart failure and calcified aortic stenosis (Boomsma et al., 1997; Boomsma et al., 2000; Altug Cakmak et al., 2015). The importance of VAP-1/SSAO for calcification of human aortic valve is also evidenced by increased VAP-1 expression in the vicinity of calcified aortic valve zones and the decreased calcification of human valvular interstitial cells by SSAO inhibition (Mercier et al., 2020). Histological examinations demonstrated that VAP-1 is highly expressed in vessels of ischemic hearts with dense leukocyte infiltrations (Jaakkola et al., 2000). This contrasts with the reduction of VAP-1 expression in vessels of ischemic brain (Airas et al., 2008), probably due to endothelial VAP-1 shedding by microglial activation and matrix protease generation in acute ischemic stroke (del Zoppo et al., 2007). Interestingly, higher basal plasma activity of VAP-1/SSAO in the acute phase of ischemic stroke predicts the increased incidence of intracranial hemorrhage after intravenous infusion of tissue plasminogen activator (tPA) to restore brain perfusion (Hernandez-Guillamon et al., 2010). Likewise, neurological outcomes of intracerebral hemorrhage are predicted by plasma VAP-1 activities as well (Hernandez-Guillamon et al., 2012). In addition, sVAP-1 is associated with major adverse cardiovascular events (MACE) and mortality in people aged >50 without prior MACE and in patients with type II diabetes (Li et al., 2011; Aalto et al., 2014).

**TABLE 1:** sVAP-1 in human cardiovascular diseases.

Disease	Ethnic group of study	Numbers of study population	Mean age, sex	Main findings	Ref
Coronary heart disease (CHD)	Taiwan Chinese	CHD^+^: N = 127, CHD^−^: N = 53	CHD^+^: Mean age = 61.5, female/male (18/109); CHD^−^: Mean age = 57.9, female/male (22/31)	Patients with CHD showed higher plasma VAP-1 levels than healthy controls	Wang et al. (2018b)
Arterial stiffness	Beijing Chinese	Healthy: N = 568	Mean age = 50.7, female/male (210/358)	Plasma sVAP-1, increased with age, was associated with arterial stiffness in subjects of age≥60	Chen et al. (2015)
Calcific aortic valve stenosis	Turk	Mild: N = 54 Mod: N = 58 Severe: N = 56	NA	Plasma VAP-1 levels consistently increased with severity of calcified aortic valve stenosis	Altug Cakmak et al. (2015)
Congestive heart failure	Dutch	CHF: N = 271 Control: N = 77	CHF: Mean age = 68, female/male (62/209); Control: Mean age = 62, female/male (28/49)	Plasma SSAO activity increased with severity of congestive heart failure	Boomsma et al. (1997)
Chronic heart failure	European	Decd: N = 195 Alive: N = 177	Decd: Mean age = 69, female/male (116/79); Alive: Mean age = 66, female/male (102/75)	Elevated plasma SSAO activity was associated with increased mortality in patients with chronic heart failure	Boomsma et al. (2000)
Ischemic stroke (IS)	Finnish	IS: N = 6 Control: N = 6	Stroke: Mean age = 60; Control: Mean age = 59; female/male (NA)	Acute stroke patients had higher levels of sVAP-1 when compared with age- and sex-matched controls	Airas et al. (2008)
	Spanish	IS: N = 48 Control: N = 92	HT: Mean age = 71.9, female/male (26/22); Control: Mean age = 70.2, female/male (38/54)	The incidence of hemorrhage formation after tPA treatment was predicted by higher basal plasma levels of VAP-1/SSAO activity	Hernandez-Guillamon et al. (2010)
Intracerebral hemorrhagic stroke (ICH)	Spanish	ICH:N = 66	ICH: Mean age = 68.8; female/male (42/24)	Plasma VAP-1/SSAO activity increased in ICH and predicted neurological outcome	Hernandez-Guillamon et al. (2012)
Subclinical atherosclerosis	Caucasian	DM: N = 29 Control: N = 25	DM: Mean age = 56.6, female/male (15/14); Control: Mean age = 52.7, female/male (14/11)	Plasma VAP-1/SSAO activity showed positive correlations with carotid plaque crouse score, total cholesterol level, and age-corrected intima-media thickness in controls	Karádi et al. (2002)
	Taiwan, Chinese	N = 115	Female mean age = 55.4, male mean age = 57.3; female/male (64/51)	Serum SSAO/VAP-1 elevation after glucose loading correlated independently to carotid IMT.	Li et al. (2009)
	Young Finnish	N = 2,182	Age range:30–45, female/male (1,199/983)	The correlations of sVAP-1 activity with cardiovascular risk factors differed between men and women	Aalto et al. (2012)
	Beijing Chinese	N = 834	Mean age = 49.1, female/male (303/531)	sVAP-1 concentration correlated with cardiovascular risk factors and subclinical atherosclerosis in an age-, sex-, and glucose-dependent manner	Chen et al. (2016)
Primary hypertension (PH)	Polish	PH: N = 121 Control: N = 28	PH: Mean age = 56, female/male (66/55); Control: Mean age = 51.5, female/male (17/11)	Plasma VAP-1 levels were elevated in PH patients with echocardiographic alterations	Maciorkowska et al. (2015)
Major adverse cardiovascular events (MACE)	Finnish	MACE: N = 265 Non-MACE: N = 2,262	MACE: Mean age = 62, female/male (89/176); Non-MACE: Mean age = 59, female/male (1,237/1,025)	sVAP-1 predicted incidence of MACE in people aged >50 without prior MACE.	Aalto et al. (2014)
Cardiovascular diseases	Taiwan Chinese	Alive: N = 501 Decd: N = 160	Alive: Mean age = 60.2, female/male (255/246); Decd: Mean age = 67.4, female/male (79/81)	sVAP-1 predicted 10-years cardiovascular mortality	Li et al. (2011)

NA: data not found; Mod: moderate; Decd: Deceased, DM: diabetes mellitus.

Clinical studies have demonstrated that serum sVAP-1 is a biomarker for atherosclerotic cardiovascular diseases (Table 1). Notably, the associations of sVAP-1 with cardiovascular risk factors and subclinical atherosclerosis are influenced by age, sex, and glucose. In Finnish subjects aged from 30 to 45, men had slightly higher levels of sVAP-1 than women, while opposite changes were observed in Chinese aged >40 (Aalto et al., 2012; Chen et al., 2016). As estrogen levels decline with age in females, higher concentrations of sVAP-1 correspond to low levels of estradiol in Chinese women. Moreover, the use of oral contraceptives in Finnish young women had a negative correlation to sVAP-1 activity, which may explain why levels of sVAP-1 were low and not correlated with age in Finnish women. It thus appears that chronic low-grade inflammations with aging and menopause lead to the elevation of sVAP-1 in women. Interestingly, a negative correlation of sVAP-1 with body mass index (BMI) has been observed in both Finish young and Chinese elder women, suggesting the increase of sVAP-1 may prevent the obesity in females. This anti-obese effect of VAP-1 seems to be estradiol-independent as they are negatively correlated. Rather, it is more likely to be attributed to the release of adipocyte VAP-1, which not only rises sVAP-1 but also reduces its insulin-mimic effects on the differentiation and lipogenesis of adipocytes. Why this effect is absent in males and whether androgen interferes with the generation of sVAP-1 are still perplexing. Notably, in young Finnish, but not elder Chinese, women, sVAP-1 represents an independent determinant of carotid intima-media thickness (IMT) and plaques. By contrast, despite no correlation with glucose in normoglycemic persons, sVAP-1 shows a strong positive association with type 1 and 2 diabetes, and independently determines carotid atherosclerotic plaques in hyperglycemic Chinese (Karádi et al., 2002; Aalto et al., 2012; Chen et al., 2016). Together, all these findings indicate that VAP-1 is involved in the pathogenesis of atherosclerosis.

## VAP-1: An Important Player in Atherosclerosis

**FIGURE 1:**
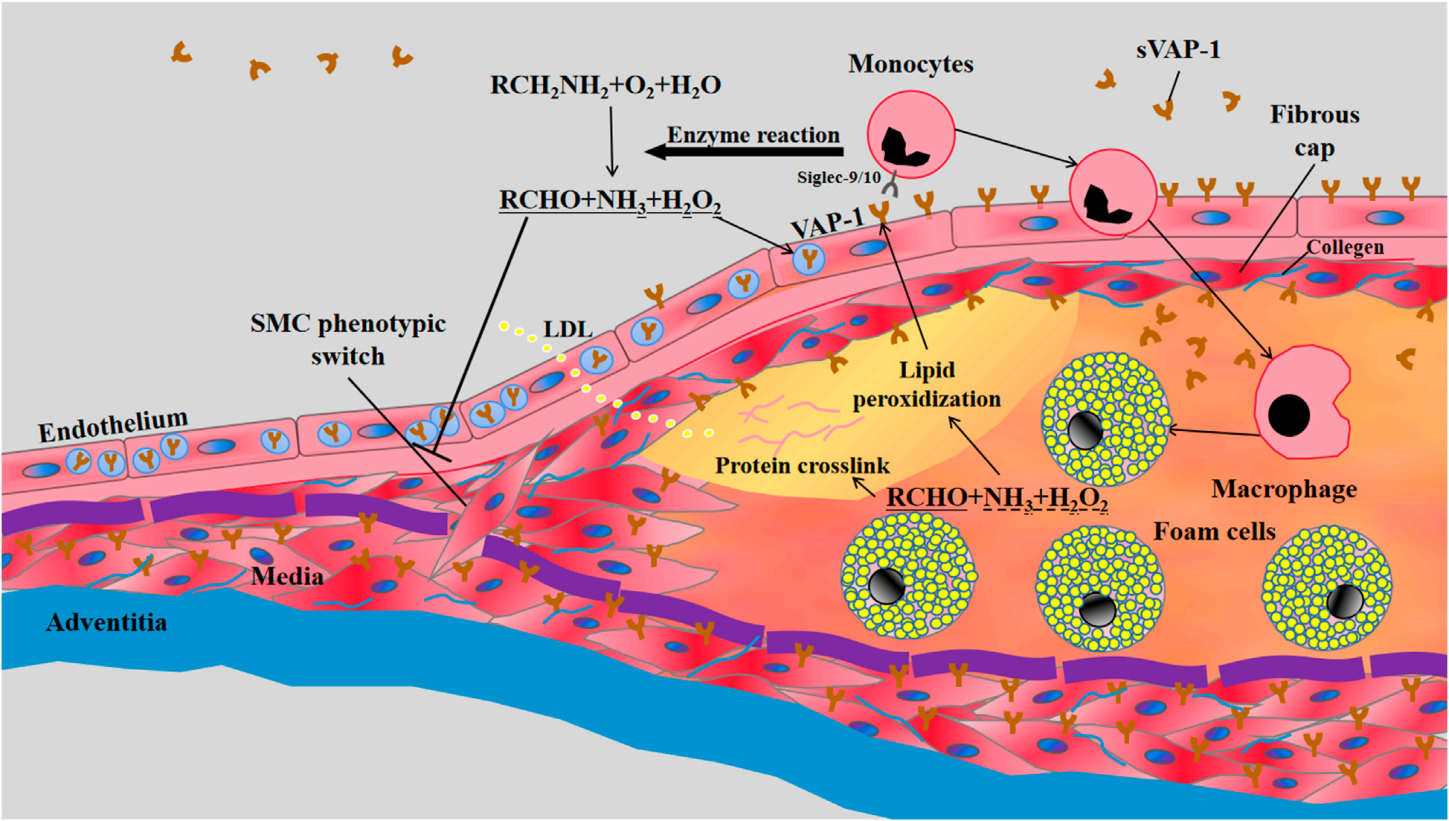
**The involvement of VAP-1 in atherosclerosis**. LDL retention and endothelial dysfunction are the essential initiators of atherosclerosis. The vascular inflammation induces the translocation of VAP-1 onto the luminal surface of endothelium, which subsequently interacts with Siglec-9/10 (sialic acid-binding immunoglobulin-like lectin 9/10) to mediate the infiltrations of monocytes into atherosclerotic lesions. Meanwhile, enzymatic products of VAP-1, such as H_2_O_2_, may further increase the expression of VAP-1 on endothelium and the subsequent transmigration of monocytes. The inflammatory early lesions stimulate the transformation of SMCs in media from the quiescent “contractile” phenotype state to the active “synthetic” state with increased proliferation, migration, and collagen synthesis. VAP-1 in the plasma membrane of SMCs may inhibit their phenotypic switch by its enzymatic products and thereby limit the growth of fibrous cap composed of SMCs and collagens. VAP-1 thus represents a pathological factor for plaque instability characterized by thin fibrous cap overlying a large lipid core rich in mon/macrophage-derived foam cells.

Atherosclerosis is a vascular inflammation caused by intima lipid accumulation and endothelial cell dysfunction. In humans, fatty streaks appear in the thoracic and abdominal aortas early in childhood, and start to develop in coronary arteries in the second decade (McGill et al., 2000). In healthy aortas, endothelium does not express VAP-1 and the high expression of VAP-1 is confined to SMCs in media (Salmi et al., 1993). Similarly, *in-vivo* VAP-1 antibody labeling experiments showed the absence of VAP-1 in the aortas of healthy C57BL/6N mice. However, the expression of VAP-1 is induced in endothelial cells lining the atherosclerotic plaques of LDLr^−/−^ApoB^100/100^ mice on 4-month Western-type diet (WTD), indicating the involvement of endothelial VAP-1 in leukocyte recruitment to atherosclerotic lesions (Silvola et al., 2016) (Figure 1). Accordingly, contents of activated macrophages in lesions were correlated with the local intensity of VAP-1 in PET imaging using [^68^Ga]DOTA-Siglec-9 (Silvola et al., 2016). Given the enrichment of VAP-1 in caveolae of the plasma membrane of SMCs (Salmi and Jalkanen, 2019), it is conceivable that the synthetic phenotype switch of SMCs, which reduces the formation of caveolae (Thyberg, 2002), will result in decreased VAP-1 expression in atherosclerotic lesions. Nonetheless, effects of hyperlipidemia and local vascular inflammatory reactions on VAP-1 expression in vascular SMCs are still unknown.

The role of VAP-1 in atherosclerosis was first disclosed in transgenic mice overexpressing hVAP-1 on endothelial cells (Stolen et al., 2004a). Compared to control counterparts, endothelial hVAP-1 overexpression did not induce more lesion formations in aortas. Instead, numbers of lesions in transgenic mice were significantly reduced, but the size of each lesion was larger than that in control mice. VAP-1/SSAO in arterial wall may generate hydrogen peroxide, aldehyde, and ammonium. These products are involved in lipid peroxidization, advanced glycation end products (AGEs) generation and protein crosslinking, all of which may lead to endothelial activation and VAP-1 induction. Moreover, higher levels of aorta SSAO and increased turnover of its substrate methylamine were correlated with increased atherosclerosis susceptibility in C57BL/6 mice (Yu and Deng, 1998). VAP-1/SSAO enzymatic reaction is thus thought to be atherogenic by facilitating leukocyte infiltration and vascular damage (Figure 1).

Recently, effects of SSAO inhibitors on the development/progression of atherosclerosis in rodents have been investigated, and findings in these pharmacological studies suggest a pathological role of VAP-1/SSAO activity in atherosclerosis (Supplementary Table 1). By using a classical SSAO inhibitor SCZ, we found that inactivation of VAP-1/SSAO promoted the stability of atherosclerotic lesions with less macrophages but more SMCs and collagen accumulations in LDLr^−/−^ mice on WTD, albeit the ineffectiveness on plasma cholesterol levels and body weight (Zhang et al., 2016). Notably, beyond reducing leukocyte infiltration, VAP-1/SSAO inactivation promotes the synthetic phenotype switch of vascular SMCs, which enhances plaque stability, both *in vivo* and *ex vivo*. Moreover, stabilization of established lesions in LDLr^−/−^ mice after lipid lowering was further enhanced by SCZ, accompanied by increased amounts of synthetic SMCs in lesions as well (Peng et al., 2016). These results indicate that the enzymatic products of VAP-1 may promote plaque vulnerability *via* SMCs (Figure 1). By contrast, in LDLr^−/−^ApoB^100/100^ mice on WTD, Sivola et al. demonstrated that 4-weeks treatment with JLP-1586, another VAP-1 inhibitor, had no effect on plaque size, body weight, or plasma lipid levels, but decreased the density of macrophages in atherosclerotic lesions (Silvola et al., 2016). In addition, Wang et al. found that ApoE^−/−^ mice or rabbits treated with PXS-4728A, a specific SSAO inhibitor, exhibited reduced atheroma with less oxidative stress and endothelial dysfunction, and markedly alleviated plasma levels of lipids and glucose (Wang et al., 2018a; Wang et al., 2018b). Given that comparable LDL-C/glucose lowering by atorvastatin resulted in a similar reduction of lesions, it seems that the atheroprotective effect of PXS-4728A is largely attributed to the reduced levels of LDL-C/glucose. Recent studies indicate that hepatic inflammation and nonalcoholic fatty liver disease (NAFLD) may result in hyperglycemia and hyperlipidemia, respectively (Min et al., 2012; Okin and Medzhitov, 2016). VAP-1 mediates hepatic inflammation of NAFLD and its inhibition modifies hepatic steatosis (Weston et al., 2015; Shepherd et al., 2020). Whether, and to which extent, the amelioration of hepatic steatosis and inflammation contributes to LDL-C/glucose lowering of PXS-4728A remains to be investigated. Future investigations on high-fat/cholesterol diet fed mice with a combined deficiency of VAP-1 and LDLr^−/−^ or ApoE^−/−^ will provide direct evidence for the role of VAP-1 in cholesterol/glucose metabolism. Impacts of VAP-1 inhibitors on plasma LDL-C/glucose levels in VAP-1^−/−^ApoE^−/−^ or VAP-1^−/−^LDLr^−/−^ mice would help to clarify their target specificity. Given the different lipid/glucose metabolisms as well as inhibitor selectivity between small rodents and humans, LDL-C/glucose lowering effects of different VAP-1 inhibitors should also be tested/confirmed in non-rodent large animals and primates. Clearly, studies on atherosclerotic lesion development in total-body and/or cell-specific VAP-1 knockout mice are still lacking to further corroborate its roles in atherosclerosis.

## VAP-1 Inhibitors: A Class of Potential Drugs for CVD

Obesity and diabetes are the major health risks associated with atherosclerotic CVD. Modulations of SSAO activity have been proven to be effective in management of (diabetic) obesity associated with low-grade adipose inflammation (Jargaud et al., 2020). Consequently, adipose inflammation and obesity are remarkably diminished by all kinds of SSAO inhibitors in obese and diabetic rodent models (Supplementary Table 1) (Papukashvili et al., 2020). Interestingly, despite a moderate and long-lasting elevation of serum glucose, the development of atherosclerotic lesions and obesity in diabetic KKAy mice was prevented by two different inhibitors, MDL-72974A, and aminoguanidine, indicating that simple hyperglycemia is not harmful for obesity and atherosclerosis in the absence of VAP-1/SSAO activity (Yu et al., 2002; Yu et al., 2004). However, the exact contribution of adipose SSAO inactivation to obesity-/diabetes-related vascular dysfunction and atherosclerotic lesion development remains unclear.

As outlined above, VAP-1/SSAO inhibitors may prevent adverse clinical events in CVD by reducing the lesion size/inflammation and/or increasing plaque stability. Moreover, VAP-1/SSAO inhibitors have shown beneficial effects on the treatment of CVD, such as stroke and myocardial infarction after onset, as well. In a rat embolic stroke model treated with tPA, SCZ prevented adverse effects caused by delayed tPA administration, leading to a smaller infarct volume (Hernandez-Guillamon et al., 2010). The administration of two VAP-1 inhibitors, LJP-1586, and SCZ, at 1 h after induction of intracerebral hemorrhage significantly reduced brain inflammation and edema as well as neurobehavioral deficits (Ma et al., 2011). Even at 6 h post-subarachnoid hemorrhage, administration of LJP-1586 improved the neurological outcome by 25% (Xu et al., 2014). Additionally, myocardial SSAO activity was increased in myocardial ischemia-reperfusion injury, and administration of SCZ, hydralazine, or LJP-1207, reduced the myocardial infarct size *in vivo* (Yang et al., 2011).

So far, clinical trials investigating hVAP-1-targeting therapeutics on CVD have not yet been initiated. Most inhibitors tested in preclinical studies could not proceed into clinical trials because of potential off-target toxicity or the appearance of better substitutions (Vakal et al., 2020). Nonetheless, PXS-4728A was safe and showed a long-lasting inhibitory effect in phase I and IIa clinical trials in NASH (non-alcoholic steatohepatitis). Unfortunately, this clinical investigation was reported to be discontinued due to the potential harmful interaction of PXS-4728A with a drug used by NASH patients (Vakal et al., 2020). Notably, a phase IIa trial of PXS-4728A in diabetic nephropathy is still ongoing. Analyzing its effect on atherosclerotic risk factors in these clinical studies will provide more insights into the therapeutic potentials of VAP-1 inhibition for CVD.

## Conclusions and Future Perspectives

SSAO/VAP-1 is involved in the development of atherosclerosis. The enzymatic activity of VAP-1/SSAO facilitates the transendothelial migration of leukocytes, while the toxic products generated may inhibit the phenotypic switch of vascular SMCs. Extensive clinical studies have demonstrated the potential value of sVAP-1 in diagnosis and prognosis of CVD. Moreover, prevention of atherosclerosis and obesity, as well as the improvement of infarcts and injury after onset of CVD are evident in rodents treated with VAP-1 inhibitors. However, cell/stage-specific effects of VAP-1 and impacts of its inhibitors on atherosclerosis, as well as underlying mechanisms, warrant further investigations in rodents and non-rodent animals.
