# Supplementary material for: Vascular Adhesion Protein-1 (VAP-1)/Semicarbazide-Sensitive Amine Oxidase (SSAO): A Potential Therapeutic Target for Atherosclerotic Cardiovascular Diseases
Source: Front Pharmacol. 2021 Jul 8;12:679707. doi: 10.3389/fphar.2021.679707 (PMC8312380; doi:10.3389/fphar.2021.679707)
Supplement: Supplementary file 1 [file Table1.pdf]

Supplementary Table 1. The inhibition of VAP-1 in animal CVD models.

Name of SSAO/ VAP-1 Inhibitors		Off Target	Model	Species	Major effects	Ref.
Allyl-amines	LJP-1586	MAO-A/B	ICH<br>SAH<br>Atherosclerotic plaque	CD1 mice<br>Sprague-Dawley rats<br>$LDLr^{-/-}$ $ApoB^{100/100}$ mice	Improved neurological scores.<br>Improved neurological outcomes.<br>Decreased density of macrophages in atherosclerotic lesions.	(Ma et al., 2011)<br>(Xu et al., 2014)<br>(Silvola et al., 2016)
	MDL-72974A	MAO-B	Atherosclerosis<br>Obesity	KKAy mice	Reduction of weight gain and atherosclerotic lesions.<br>Reduction of weight gain.	(Yu et al., 2002)<br>(Yu et al., 2004)
	PXS-4728A	/	Atherosclerosis	New Zealand white rabbits<br>Apo $E^{-/-}$ mice	Reduction of weight gain and atherosclerotic plaques.<br>Reduction of atheroma and oxidative stress.	(Wang et al., 2018a)<br>(Wang et al., 2018b)
Hydra-zines	Aminoguani dine	DAO	Atherosclerosis	KKAy mice	Reduction of weight gain and atherosclerotic lesions.	(Yu et al., 2002)
	Phenylhydra zine	MAO-B	Obesity	Zucker rat	Reduction of weight gain.	(Carpéné et al., 2019)
	SCZ	LO	Embolic stroke<br>Atherosclerosis<br><br><br>MI<br>ICH	Sprague-Dawley rats<br>$LDLr^{-/-}$ mice<br><br><br>Sprague-Dawley rats<br>CD1 mice	Reduction of the infarct volume.<br>Decreased macrophages/increased SMC in established lesions after treatment with/without lipid lowering.<br>Decreased macrophages/increased SMC in atherosclerotic lesions during lesion formation.<br>Reduced infarction sizes.<br>Improved neurological scores.	(Hernandez-Guilla mon et al., 2010)<br>(Peng et al., 2016)<br>(Zhang et al., 2016)<br>(Yang et al., 2011)<br>(Ma et al., 2011)
	LJP-1207	/	MI	Sprague-Dawley rats	Reduced infarction sizes.	(Yang et al., 2011)
	Hydralazine	MAO-A/B	MI	Sprague-Dawley rats	Reduced infarction sizes.	(Yang et al., 2011)
VAP-1 siRNA			ICH	CD1 mice	Improved neurological scores.	(Ma et al., 2011)

Note: All above inhibitors, except VAP-1 siRNA, are irreversible inhibitors that bind to the topaquinone (TPQ) cofactor of VAP-1.
MAO-A: monoamine oxidase A; MAO-B: monoamine oxidase B; ICH: intracerebral hemorrhage; SAH: subarachnoid hemorrhage; MI: myocardial infarction; DAO: diamine oxidase; LO: lysyl oxidase; SCZ: Semicarbazide;
